# Professorial Changes and Appointments

**Published:** 1850-05

**Authors:** 


### PROFESSORIAL CHANGES AND APPOINTMENTS.

Dr. N. Chapman has resigned the chair of the Theory and Practice of Physic in the University of Pennsylvania, and has been appointed Emeritus Professor.

For more than forty years, have large and admiring classes listened to the teachings of Professor Chapman. Called, when yet under twenty years of age, to the responsibilities of a public teacher, as assistant to Doctor James, then Professor of Obstetrics, he soon succeeded in gaining the confidence of those who entrusted themselves to his guidance. In 1813 he was appointed Professor of Materia Medica, and in 1816 he succeeded the late Dr. B. S. Barton as Professor of the Theory and Practice of Physic. To all who are familiar with his worth—and who is not?—this announcement will be heard with regret, but the feeling will be mingled with satisfaction that the evening of his days has found him crowned with the brightest honors of the profession, and rich in the affections and gratitude of all who know him.

---

Dr. Landon C. Rives has been appointed Professor of Materia Medica in the Medical College of Ohio, to fill the vacancy caused by the death of the lamented Harrison.

---

We have received from Mr. George D. Phelps, of New York, specimens of several articles of value to the profession. Among these is the Cantharidine blistering plaster and dressing, with which our readers are doubtless familiar as a new and exceedingly neat preparation, easy of application and certain in its effects. We have given it a trial, and find it fully to answer our expectations.

We are under obligations to the same house for specimens of

Lawrence's hair gloves, brushes, &c., for external friction. A limited trial of them has satisfied us of their admirable fitness for the uses for which they are intended. We refer our readers to the advertisement in this number, in which they will find all the information respecting them which they may desire.

